# Optimisation of Backing Layer Formulations via Rational Polymer Selection to Improve the Insertion of Dissolving Microneedles Into Skin

**DOI:** 10.1021/acs.molpharmaceut.5c01024

**Published:** 2026-01-09

**Authors:** Fiona Smith, Benjamin Fiedler, Khaled Elkassas, Ruslan Mohamed, Karmen Cheung, Mischa Zelzer, Abina Crean, Faz Chowdhury, Joel Segal, Frankie Rawson, Maria Marlow

**Affiliations:** a School of Pharmacy, 6123 University of Nottingham, Nottingham NG7 2RD, United Kingdom; b School of Pharmacy, 8795 University College Cork, Cork T12 YT20, Republic of Ireland; c Advanced Technology Centre, 383065 Nemaura Pharma Limited, Oakwood Drive, Loughborough, Leicestershire LE11 3QF, United Kingdom; d Department of Mechanical, Materials and Manufacturing Engineering, Faculty of Engineering, 6123 University of Nottingham, Nottingham NG8 1BB, United Kingdom

**Keywords:** microneedles, skin, material properties, insertion efficiency, backing layer

## Abstract

Dissolving microneedles (MNs) hold promise as a versatile drug delivery platform, particularly suited to the delivery of complex molecules across the skin. Dissolving MNs are commonly manufactured using an accessible and reproducible two-step casting process. The selection of different polymers for both the needle and backing layer increases the adaptability of this platform. Previously, work has focused on the needle layer formulation and how the formulation will affect drug delivery. Less well understood is the role of the backing layer on insertion and, subsequently, drug delivery. Therefore, the aim of this work was to evaluate changes to the backing layer formulation on MN insertion and understand the relationships between material properties. The needle layer was formulated with polyvinylpyrrolidone-*co*-vinyl acetate, with and without insulin, a model protein therapeutic. A range of polymers was used to formulate the backing layer, including sodium carboxymethylcellulose (Na-CMC), poly­(vinyl alcohol) (PVA), and polystyrene (PS). MNs manufactured with a PVA backing layer demonstrated an improved insertion profile (efficiency and depth). Permeation studies supported that the PVA backing layer offered an overall advantage in insulin delivery, with a cumulative recovery of 17.6% of the total insulin loading. This work demonstrates the importance of the backing layer formulation in MN arrays. Changing the backing layer formulation impacted both the insertion of MNs and subsequent drug delivery. Moving forward, the properties of polymers selected for use in MN backing layers should be thoroughly explored and rationally selected depending on the intended application.

## Graphical abstract

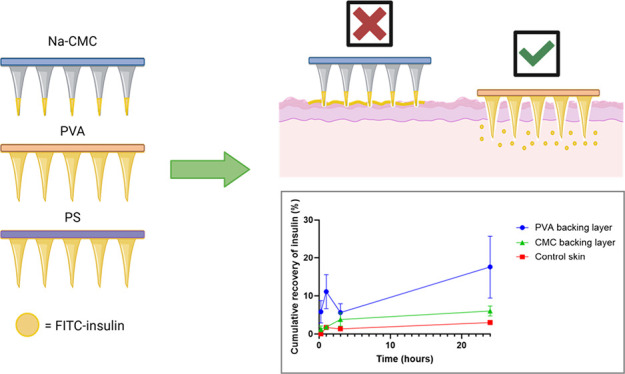

## 1.0 Introduction

Microneedle (MN) arrays continue to hold promise as a drug delivery platform that can overcome physiological barriers, including skin, impermeable to complex and large molecules. Several different classes of MN remain under investigation by researchers, such as hydrogel, hollow, solid, and dissolving.^1^

Dissolving MNs have attracted attention owing to their self-disabling nature post -administration, their reasonable drug-loading capacity and patient-friendly nature. Typically, dissolving MNs have been made from a casting process, which is both reproducible and technically simple.^1^ Solutions of polymers are cast into molds and allowed to dry, either at room temperature, elevated temperature or under vacuum. Owing to the ability to add different solutions into the mold over time, MN arrays incorporating multiple materials can be designed and manufactured. Most commonly, this is demonstrated by the selection of a different polymer for the needle layer (NL) and backing layer (BL).

Several different backing layer formulations have been identified in the literature, with certain polymers featuring commonly. These include sodium carboxymethylcellulose (Na-CMC), poly­(vinyl alcohol) (PVA), polyvinylpyrrolidone (PVP) and poly­(acrylic acid) (PAA).^2–4^ In part, the selection of these materials relates to their safety profiles, making them ideal candidates for translational research. However, despite the range of different formulations used, there is often little explanation of the material choice beyond the biocompatibility and its previous use. Instead, focus is commonly placed on the properties of the needle layer of the MN array, which has been perceived to have the most pronounced effect on insertion.^5^

However, even with optimization of the needle layer formulation, it is commonly accepted that MN insertion fails to reach depths equivalent to the total needle length. In 2016 Loizidou et al. published the results of a study which used microCT to visualize the insertion of MNs into porcine skin.^6^ The maximum penetration recorded was 34% of the MN height (1000 μm), dependent on the MN geometry. Later, Li et al. conducted a study that compared the insertion of 18 MN arrays with different geometries into the skin of human participants.^7^ Consistently, MNs with a length of 800 μm achieved a penetration of around 90% of the needle length, which dropped to 38–59% when 1400 μm needles were used. In terms of dosing efficiency, the inability for complete insertion with the longer MNs meant that similar quantities of drug were delivered with both the longest and the shortest MNs, despite having a higher drug loading. More recently, Soorani et al. have used computational modeling to study the insertion of MNs, which was later validated with experimental work using PVP and PVA MNs.^8^ Soorani et al. showed that around 50% of the MN remained outside of the skin after a 35 N force was applied to the backing layer. The incomplete insertion presents a particular issue when drug is loaded homogeneously throughout the needle layer. Without full insertion into the skin, it is unlikely that the total payload will be delivered. This is likely to affect drug plasma concentrations and could lead to concerns from regulatory agencies.

Few studies have explored how the backing layer formulation may affect insertion and drug release. Chellathurai et al. completed a study in which the amount of plasticizer and the ratio of PVA:PVP were varied within the backing layer of the MNs.^9^ The addition of a plasticizer was essential for good flexibility and successful removal from the glass slide they were cast onto. As anticipated, with the increase of PEG 400 the tensile strength and percentage elongation increased. Research conducted by Loizidou et al. explored three different MN formulations produced with CMC and either maltose, trehalose or sucrose, manufactured in a one-step process.^10^ Penetration studies confirmed differences in the maximum penetration depth measurable when the three formulations were tested. Although not explored during the study, it should be considered whether changing the composition of the backing layer contributed to changes in the MN insertion. Meanwhile, Yan et al. explored the combination of CMC with different ratios of PVP, hyaluronic acid (HA) and PVA.^11^ Extensive testing of the mechanical properties of the formulations, including insertion, were conducted. The material with the highest tensile strength was selected as most favorable, owing to the expectation that it would be most suited to skin penetration. The design of this study, among others, highlights the lack of direct testing on the material choice for the backing layer. As such, it cannot be stated with certainty that the backing layer material choice does or does not affect the penetration of the MNs in the skin. While previous work has frequently explored the needle layer formulation, use of applicators and the skin topology, there is a clear knowledge gap in understanding the role of the backing layer on insertion.

The work presented here aimed to explore the role that the backing layer formulation had on the insertion of MNs and the subsequent transdermal delivery of a therapeutic by formulating MNs with a range of different polymer backing layers. In keeping with the literature, Na-CMC was selected for its favorable water solubility and established use in pharmaceuticals. Previously, Na-CMC films have displayed flexibility and toughness that may favor the use as a backing layer.^12^ PVA was selected as a second water-soluble polymer. PVA has also previously been well-utilized and characterized in pharmaceuticals and, more specifically, MN research.^13–15^ Finally, PS, a common hydrophobic polymer, was selected, having previously demonstrated it can successfully form a backing layer.^3^ Throughout this work, insulin was used as a model protein owing to its high molecular weight and hydrophilic structure, making it an ideal candidate for transdermal delivery via MNs.

It was hypothesized that different polymer formulations of the backing layer may have a significant impact on MN insertion. The findings of this study are particularly relevant to researchers working in transdermal drug delivery or the clinical translation of MNs.

## 2.0 Experimental Section

### 2.1 Materials

Glycerol, polyethylene glycol (PEG 400), poly­(vinyl alcohol) (Mw 30–70 kDa), sodium carboxymethyl cellulose Mw 90000, insulin solution human and Parafilm M were purchased from Sigma-Aldrich. Polyvinylpyrollidone-*co*-vinyl acetate (PVPVA, Kollidon VA 64 Mw 45–70 kDa) was provided by BASF. Lyophilized human recombinant insulin was purchased from Merck Life Sciences Ltd. Fluorescein isothiocyanate (FITC) was purchased from Fluka Biochemika. HPLC grade (ultrapure) water (18.2 MΩ.cm) was available from a SLS Lab Pro PURA-Q+20 Type 1. Teepol solution was purchased from Scientific Laboratory Supplies. Gentian violet 1% w/v was purchased from De La Cruz products. OCT compound was purchased from VWR International Ltd. Human/canine/porcine insulin DuoSet ELISA and ancillary reagent kits were purchased from R&D systems, Biotechne. Silicone elastomer (Sylgard 184) and Silicone elastomer curing agent (Sylgard 184) were purchased from Wiesbaden, Germany. Fresh, untreated porcine skin was procured from Outwood Farm, Cheadle. The full-thickness skin was immediately washed, cut, packaged and stored at −20 °C prior to use.^16^ To minimize changes to the biomechanical properties of the skin, care was taken to ensure skin was not exposed to repeated freeze–thaw cycles.

### 2.2 Methods

#### 2.2.1 Manufacture of Blank MN Arrays Using a Range of Polymers

Stainless steel 304 master molds with a 12 mm × 12 mm square base (base depth 300 μm) with 10 × 10 obelisk MNs protruding to a height of 1000 μm, (pitch 800 μm) were made using a Kern Evo CNC micromilling machine at the Precision Manufacturing Centre, University of Nottingham.

Thereafter, PDMS molds were fabricated using a 10:1 ratio of silicone elastomer to silicone curing agent (Sylgard 184), which was mixed thoroughly prior to being degassed. 1.25 mL of the mixture was transferred to the stainless steel 304 master mold and held under vacuum for 5 min or until air bubbles migrated to the surface. After release from the vacuum, molds were cured at 90 °C for 30 min before being placed in an ice bath and removed from the stainless steel mold.

Dissolving MNs were made using a two-step casting process, visualized in Figure SI1. First, the needle layer was manufactured using 16.2% w/v polyvinylpyrollidone-*co*-vinyl acetate (PVPVA) and 2% v/v polyethylene glycol (PEG) 400 in water. 150 μL solution was pipetted into a mold and centrifuged at 4000 rpm for 15 min (Heraeus Multifuge 3s, Kendro Laboratory, Germany). Once centrifuged, the molds were tilted such that excess liquid gathered and could be removed using a pipette, leaving only the channels filled to form the needles. The molds were placed in a desiccator overnight to allow the needle layer to dry.

The backing layer was originally made using 5.2% w/v CMC and 0. 65% v/v glycerol in water. 200 μL solution was pipetted directly on top of the needle layer and further centrifuged at 3500 rpm for 10 min. The molds were placed in a desiccator and left to dry for 72 h. After this time, the MNs were carefully removed from the molds. The MNs were left for a further drying period within the desiccator and stored there until use. Later, two further backing layer formulations were tested; an aqueous 20% w/v PVA solution and 20% w/v PS dissolved in 1,4-dioxane (placed under vacuum).

For the three point bending test (section 2.2.12), backing layers only (without microneedles) were made using a PDMS mold with the same geometry and volume as the MN array backing layer. 200 μL of the polymer backing layer solutions were pipetted into the PDMS molds. The molds were then centrifuged at 3500 rpm for 10 min to ensure that the solution filled the cavity of the PDMS mold. The filled PDMS molds were then dried at 30 °C overnight before being stored in a desiccator at ambient temperature and left to dry for 72 h, before being demolded and stored under the same conditions prior to use.

#### 2.2.2 Insulin Reconstitution

Human recombinant insulin was reconstituted to a concentration of 20 mg/mL.^17,18^ Insulin was weighed out, then 120 μL of 0.1 M hydrochloric acid was added and gently aspirated using a pipette until insulin dissolved. HPLC-grade water or deuterium oxide (heavy water) was added to make the final volume to 1 mL. Successful reconstitution was verified using FT-IR and circular dichroism.^19^

#### 2.2.3 Synthesis of FITC Insulin

FITC was conjugated to insulin and analyzed using the same methods as previously published.^19^ The FITC-insulin was stored in a −20 °C freezer until use.

#### 2.2.4 Manufacture of Insulin-Loaded MN Arrays

Reconstituted insulin was incorporated into the MN arrays during the process of casting the needle layer.

Immediately prior to MN fabrication, lyophilized human recombinant insulin was reconstituted, as per Section 2.2.2. The reconstituted insulin was added to the needle layer solution, producing a 0.4% w/v solution. After drying, the resulting MN arrays had a drug loading of 24 μg, equivalent to 0.69 insulin units.

Alternatively, FITC-insulin was loaded into the MN arrays to allow visualization of insulin, replacing the standard insulin. The total insulin content and manufacturing method remained constant to reduce variation in the physical MN properties.

#### 2.2.5 Design, Manufacture, and Assembly of an Applicator for MN Arrays

An applicator was designed incorporating the body of a commercially available MN, similar to a DermaPen, with a 3D printed 1.5 cm × 1.5 cm square-based platform. The part was designed on SolidWorks 2019 (Dassault Systèmes) and printed using VeroWhite Plus (RGD835) and Support (SUP705) on an Objet30 Prime, Sys UK.

Using superglue, the platform was secured to a disposable solid MN array that could be attached to the MN device, eliminating the pre-existing needles. Owing to the capability of the MN device to oscillate, the applicator was also able to oscillate.

#### 2.2.6 Scanning Electron Microscopy

Low vacuum scanning electron microscopy (SEM) was carried out to evaluate the MN structure post demolding. MN devices were attached to a vertical stub using carbon adhesive tape. Images were acquired in low vacuum mode of the FEI Quanta 650 SEM using water vapor as the imaging gas and a large field detector (LFD). Operating voltage (kV) and gas pressure are displayed on the data bar for each image. MN devices were remounted flat and imaged again at 0° tilt using the same imaging conditions.

#### 2.2.7 Fluorescent Microscopy

After demolding, an EVOS M5000 imaging system was used to visualize the FITC-insulin using the green fluorescent protein (GFP) channel (482 nm excitation, 524 nm emission). Samples with untagged insulin and no insulin (blank) were imaged as controls.

#### 2.2.8 Fracture Force

A texture analyzer (TA-XT Plus Texture Analyzer, Stable Microsystems, UK) was used to evaluate the fracture force of manufactured MNs. A MN array was attached to a 20 mm cylindrical aluminum probe using double-sided tape, with the needles facing downward, and attached to a 50 kg load cell. The settings were adjusted such that a compression test was completed with a pretest and post-test speed of 10 mm/sec. The trigger force was set to auto at 0.02 N with no break mode. As the MNs met the lower aluminum block, the compression force was plotted against displacement, which was later converted to fracture force in Newtons (N) per needle by taking the highest value of compression prior to plastic deformation. A minimum of 4 repeats were taken for each MN formulation.

#### 2.2.9 *In* *V* *itro* Insertion Studies

Parafilm M was initially used as an *in vitro* model for skin to gain an understanding of how well MNs may be inserted into human skin.^20^ Eight layers of Parafilm M were stacked on top of one another and placed on a cork mat prior to having MNs inserted. MNs were pushed down using thumb pressure for 10 s and then removed. Each layer of Parafilm M was analyzed using an optical microscope (Zeta Profilometer, KLA-Tencor, US) to identify the successful formation of microchannels.

The insertion efficiency achieved was calculated using eq 1 to allow direct comparison between formulations.

$$\mathrm{Insertion}\;\mathrm{efficiency}\;(\%)\frac{\mathrm{Number}\;\mathrm{of}\;\mathrm{microchannels}\;\mathrm{formed}}{\mathrm{Theoretical}\;\mathrm{number}\;\mathrm{of}\;\mathrm{microneedles}\;\mathrm{on}\;\mathrm{the}\;\mathrm{array}}\times 100$$ 1

#### 2.2.10 *Ex* *V* *ivo* Insertion Studies

Immediately prior to use, porcine skin was defrosted at room temperature and hair removed using microscissors. Skin was placed on a cork mat while the MNs were inserted. MNs were inserted by applying thumb pressure to the backing layer for 10 s. 1% w/v gentian violet solution was immediately added to the skin after the MNs were removed and left for 1 h. Thereafter, skin samples were flash-frozen in liquid nitrogen, mounted with optimal cutting temperature (OCT) compound and cross-sectioned using a cryostat (Leica CM3050 S Research Cryostat, UK). The microchannels formed were imaged and measured using an optical microscope.

Insertion efficiency was calculated using eq 1. Quantification of the successful microchannel formation was possible due to the demarcation produced by the gentian violet stain.

The applicator developed in section 2.2.5 was tested to understand whether this may improve the insertion of the MN array. The method used remained the same other than applying pressure to the backing layer of the MN for 10 s using the applicator, rather than thumb pressure.

#### 2.2.11 Assessment of Insulin Stability

An Agilent Technologies Cary 630 FTIR spectrophotometer was used to measure the transmittance of solid and liquid samples between 650 and 4000 cm^–1^ using attenuated total reflectance (ATR). Whole MN arrays were tested owing to the difficulty in separating the needle layer from the backing layer. Agilent MicroLab software was used for data collection.

OriginLab (Pro) 2023 was used for the plotting and analysis of data.

#### 2.2.12 Three-Point Bend Testing

A texture analyzer (TA-XT Plus Texture Analyzer, Stable Micro Systems, UK) equipped with a three-point bending rig was used to test the hardness, flexibility, and toughness of microneedle backing layers. The backing layers were placed on the supports with an internal separation of 4.8 mm. The instrument was equipped with a 50 kg load cell. The pretest speed was 1 mm/sec and the post-test speed was 10 mm/sec, the trigger force was set at 25 g, and the strain rate was 10%/sec. As the bending probe met the backing layers, the compression force (g) was plotted against displacement (mm). The software calculated the hardness, flexibility, and toughness from the compression force displacement data (shown in SI2). Six repeats were conducted with each backing layer formulation.

#### 2.2.13 Adhesion Studies

The texture analyzer (TA-XT Plus Texture Analyzer, Stable Microsystems, UK) was further employed to understand the adhesion of MN arrays to skin and whether this varies with different materials.

A piece of porcine skin was prepared (defrosted and excess hair removed) and clamped to the heavy-duty platform to ensure it remained in place throughout the test. The MN array was secured to a 20 mm cylindrical aluminum probe using double-sided tape, with the needles orientated downward.

An adhesive test was selected from the texture analyzer software and modified to include the following parameters. Pretest speed 10 mm/sec, test-speed 2 mm/sec, post-test speed 0.1 mm/sec, applied force 20 N, return distance 5 mm, contact time of 60 s and an automatic trigger of 0.02 N.

Adhesion was defined as the maximum force required to remove the MN array from the skin, characterized by an immediate sharp drop in force thereafter.

#### 2.2.14 Pressure Distribution

The distribution of force applied during MN insertion was explored using pressure-sensitive indicating film (PSIF) (ultra super low pressure (LLLW) two-sheet Prescale film, Fujifilm) which was placed directly onto porcine skin (*stratum corneum* facing upward, with PSIF above this) attached to the heavy-duty platform of the texture analyzer. Then the method described above was used to lower the MNs onto the skin. The pressure-sensitive film changed color upon having force applied to it via the MNs (measurable pressure range 0.2–0.6 MPa).

#### 2.2.15 *Ex* *Vivo* Drug Release and Permeation Study

A Franz diffusion cell (FDC) study was employed to measure the permeation of insulin when administered from a MN array. Full thickness *ex vivo* porcine skin was defrosted at room temperature, excess hair was removed. Three mL of PBS and a magnetic stirrer were added into the receptor compartment of a FDC. Small squares of skin (approximately 1.5 cm × 1.5 cm) were prepared. MNs were inserted by applying thumb pressure for 10 s and applying narrow strips of autoclave tape to secure the MN in place before the skin was clamped between the receptor and donor compartments. Franz diffusion cells were placed in a water bath set to a constant temperature of 36.5 °C and a magnetic stirring speed of 840 rpm.

One mL of receptor fluid was collected after 15 min, 1 h, 3 and 24 h and immediately replaced with new PBS to maintain sink conditions. Samples were filtered with a 0.22 μm membrane prior to analysis. After 24 h the Franz cells were dismantled. Six repeats with MN insertion were completed for each formulation tested.

A human/canine/porcine insulin ELISA kit (DY8056–05, Biotechne R&D systems) was used to quantify the insulin released from the dissolving MNs. Owing to the low insulin loading capacity of the MNs, HPLC was deemed inappropriate. After collection, samples were diluted 1:1000 to ensure they contained concentrations of insulin within the calibration curve.

The method used was as follows. The capture antibody was reconstituted and diluted before being used to coat each well of a 96-well plate and left to incubate overnight. The next day, each well was washed using wash buffer, emptied then repeated three times before blotting dry. Next, plates were blocked using reagent diluent for 1 h before repetition of the washing step. The diluted samples, as well as those used for the calibration curve, were added to each well and left for 2 h before being washed out. Detection antibody was added to each well and left for a further 2 h before being washed to remove excess. Next, streptavidin-HRP B was added to each sample-containing well and incubated for 20 min before being washed out. Thereafter, the substrate solution was added and left for a further 20 min. Finally, stop solution was added to each well, turning the color yellow. Absorbance was measured using a Tecan Spark 10 M multimode plate reader at 540 and 450 nm, then subtracted from each other to allow for optical imperfections within the 96-well plate.

### 2.3 Statistical Analysis

Results were reported as mean value with standard deviation (SD) or standard error of the mean (SEM) (for biological repeats). Statistical calculations were performed using GraphPad Prism 10 software (IBM, USA). A one-way analysis of variance (ANOVA) followed by a Tukey posthoc test was applied to compare multiple data groups. When necessary, a two-way ANOVA with multiple comparisons was used to understand how independent variables may have a combined effect on the outcome. When two data sets were being directly compared, Welch’s *t* test was employed. A statistically significant difference was denoted by a p value <0.05.

## 3.0 Results and Discussion

### 3.1 Production and Physical Characterization of MN Arrays Made from a Range of Polymers

Previous work has demonstrated that PVPVA, marketed as Kollidon VA 64 by BASF, formed desirable MNs with properties suitable for insertion into skin.^21^ Additionally, PVPVA properties such as biocompatibility and solubility favored manufacturing of the needle layer of the array. Consequently, PVPVA was selected as the needle layer material for this work.

SEM was used to verify the structural uniformity of the MNs. Figures 1 (i) A) and C) (Na-CMC and PVA BLs) show MNs with well-defined geometries and sharp tips, appropriate for insertion into skin. However, Figure 1 (i) B) and E) distinctly show that MNs featuring a PS backing layer are severely deformed, with the tip rounded rather than sharp, which would prohibit piercing of the skin.

**Figure 1:**
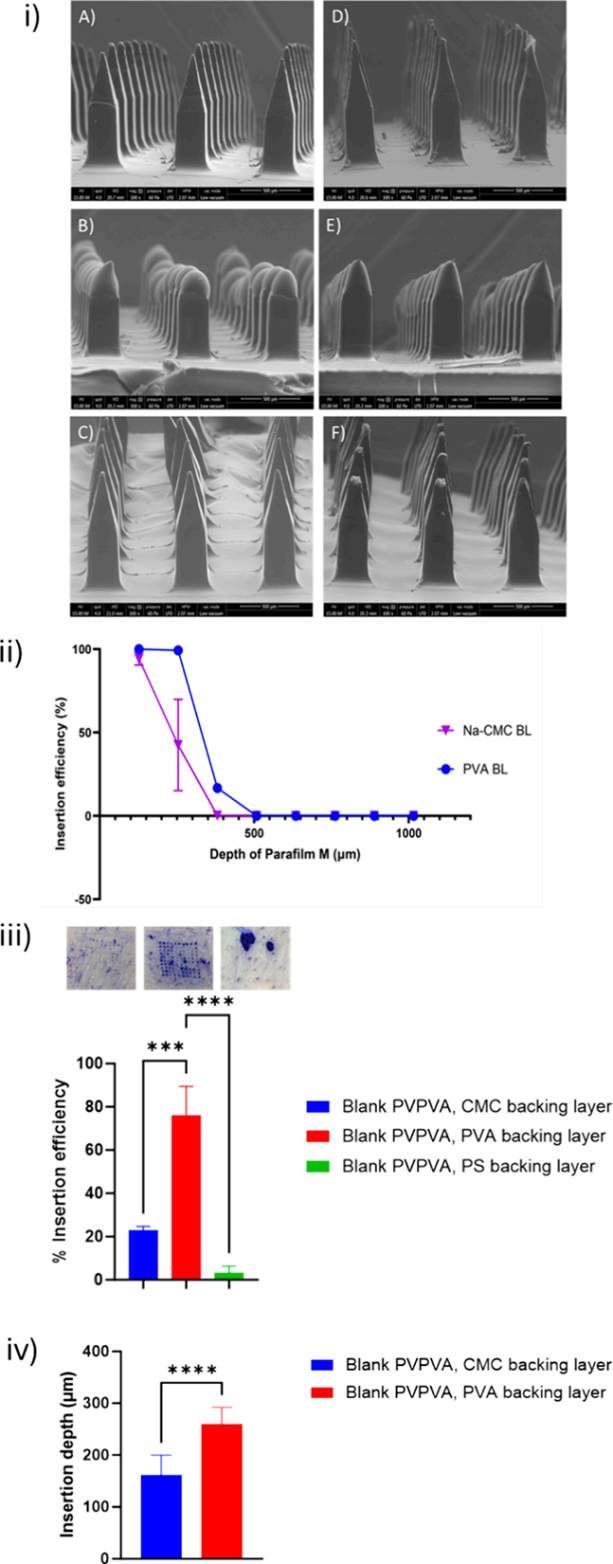
Micrographs in (i) use SEM to show the structure of blank PVPVA MNs with backing layers made from A) PVA, B) PS and C) Na-CMC and insulin loaded MNs with backing layers made from D) PVA, E) PS and F) Na-CMC, at 100× magnification. Scale bar represents 500 μm. Graph (ii) shows an in vitro study to evaluate the insertion efficiency and depth. Data expressed as mean ± SD, *n* = 4. Graph (iii) illustrates the insertion efficiency of MN arrays in ex vivo porcine skin with corresponding images of the punctuations. The PVA backing layer has statistically improved insertion compared to both the Na-CMC and PS, where *p* < 0.001 (***) and *p* < 0.0001 (****) respectively. Data expressed as mean ± SD, *n* = 3. Graph (iv) shows the insertion depth of MNs in ex vivo porcine skin. The graph shows a statistically significant improvement in insertion depth when PVA was used, *p* < 0.0001 (****). Data expressed as mean ± SD, *n* = 10.

To further understand whether the change in backing layer formulation affected the insertion profile, an *in vitro* insertion study was completed to compare blank MNs with a Na-CMC and PVA BL. Figure 1 (ii) compares the *in vitro* insertion depth of formulations. When MN arrays with a PVA backing layer were tested compared to a Na-CMC backing layer, the PVA backing layer improved the insertion efficiency at each layer of the Parafilm M up to a depth of 381 μm.

MNs with a PS BL were not tested at this stage owing to difficulties with consistent manufacturing. As well as the rounded tips, obtaining a uniform backing layer proved challenging. Commonly, bubbles were seen forming as the solvent evaporated from the backing layer solution, which disturbed the integrity of the backing layer and potentially the needle layer alike.

To build on the *in vitro* findings, the formulations were taken forward into an *ex vivo* insertion study, also shown in Figure 1. As anticipated, from Figure 1 (iii), the PVA backing layer demonstrated a significant improvement in insertion efficiency.

To understand more about the potential benefits of a PVA backing layer in terms of insertion, the *ex vivo* skin was cryosectioned and channel depth measured using the method outlined in 2.2.10. Figure 1 (iv) shows that on average the MNs with a PVA backing layer produced an insertion depth of 259.5 μm, a statistically significant improvement compared to the Na-CMC BL (*p* < 0.0001).

It is well reported that the molecular weight of a polymer directly contributes to the mechanical properties of the material.^22,23^ The main constituents used in the backing layers here, PVA, PS and Na-CMC, have high molecular weights, designed to increase the hardness of the backing layer. While this could be a contributing factor to the insertion capabilities of the MNs, no clear relationship was observed here. Given the plethora of other factors that contribute to the MN insertion, such as the MN tip diameter, a more controlled study would be desirable to explore this. Developing this further, comparing equivalent molecular weights and the subsequent adjustment to the molecular weight of the selected polymers may be beneficial to allow the rational selection of a polymer for the backing layer.

This work suggested that the material used for the backing layer formulation of MNs has a pronounced and significant effect on the insertion capabilities of the MN array. Notably, when PVA was used as the principal polymer in the backing layer, MNs were well-formed, with improved insertion efficiency and depth. While these initial findings indicated PVA to be advantageous as a backing layer, given MNs are intended to be used as a drug delivery platform, further investigation was required using drug loaded MNs. However, first the use of an applicator was evaluated as to whether this could improve insertion.

### 3.2 Evaluation of the Use of an Applicator to Improve the Insertion Profiles of MNs

Even with the improvements observed through changing the backing layer, it was hypothesized that the insertion depth and efficiency may be further improved through use of an applicator. As such, a novel applicator was created and tested using *ex vivo* insertion studies.

The applicator was designed with a square-based platform designed to cover the backing layer of the MN arrays, ensuring the whole MN patch would be in contact with the applicator and apply equal force across all MNs. The commercial MN device selected for the base of this applicator contained an electronic mechanism that facilitates oscillation. Previous work by Sabri et al. suggested oscillation may be advantageous, improving the insertion efficiency and depth.^24^ It was hypothesized that the repeated application of force directly to the backing layer of the MN array may be responsible for this.

Figure 2 (ii) shows the insertion efficiency achieved with the oscillating applicator compared to application using thumb pressure in *ex vivo* porcine skin. Unexpectedly, there is no clear advantage with the use of the applicator compared to thumb pressure. Indeed, thumb pressure significantly improves the insertion efficiency achieved with a PVA BL.

**Figure 2:**
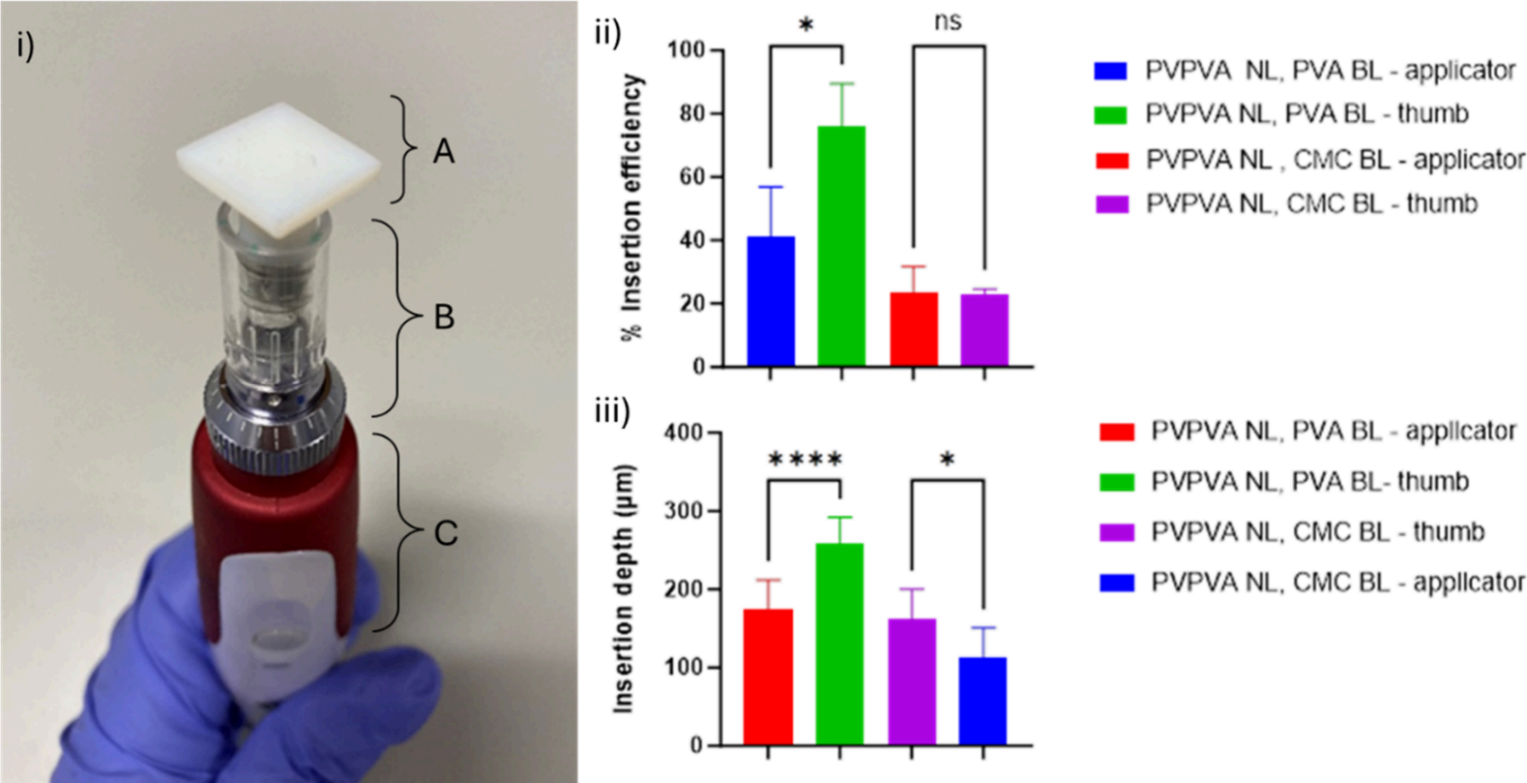
Image (i) a novel MN array applicator manufactured by attaching a square-based platform (A) to a disposable MN attachment (B) connected to an oscillating cosmetic MN device (C), producing an oscillating applicator. Graph (ii) a comparison between the insertion efficiency of insulin loaded MN arrays when an oscillating applicator compared to thumb pressure. MNs with a PVA BL has statistically improved insertion efficiency when thumb pressure is used, *p* < 0.05 (*). Data expressed as mean ± SD, *n* = 3. Graph (iii) the insertion depth of insulin loaded MN arrays in ex vivo porcine skin when an oscillating applicator is used compared to thumb pressure. The graph shows a statistically significant improvement in insertion depth when thumb pressure was used *p* < 0.0001 (****) with a PVA BL and *p* < 0.05 (*) with a Na-CMC BL. Data expressed as mean ± SD, *n* = 10.

To further explore this the insertion depth achieved when penetration successfully occurred was measured, as shown in Figure 2 (iii). Here, it is the case that the applicator has reduced the insertion depth achieved by the MNs, contrary to the hypothesis that this would be improved.

One possibility is that the force applied by the applicator during each oscillation was not enough to insert the MNs, particularly considering the distribution of force across all 100 MNs in the array. Another explanation may be related to the speed that the applicator will insert the MNs at. Previous work has determined the velocity to be a critical factor for the successful insertion of MNs in skin.^25^

As further demonstrated by Figure 2 (iii), application using thumb pressure demonstrated superior insertion, leading to this approach being used in work going forward. However, it should not be overlooked that the pressure applied between different patients would vary, potentially limiting the success of thumb application of MN patches in the clinic. In the future, significantly more development would be required to design and manufacture an applicator which improves MN insertion. Nevertheless, this further supports that the choice of backing layer remains a critical factor that can be readily adjusted and will have a noteworthy effect on insertion.

### 3.3 Production and Physical Characterization of Insulin-Loaded MN Arrays

Studies to characterize the insulin loaded MNs were completed to confirm there were no significant changes to the needle layer during the process of integrating insulin into the polymer. As expected, SEM micrographs shown in Figure 1 (i) showed that MNs appear similar in structure when insulin-loaded compared to blank. Most notably, as observed previously, the MNs with a PS backing layer continue to lose their sharp peak, suggesting these will not favor insertion.

The methodology of the fracture force test favors quantification of the strength of the needle layer specifically, rather than the backing layer, making it valuable in assessing changes related to drug loading. Testing the fracture force of insulin loaded PVPVA MNs demonstrated no statistically significant difference compared to the blank PVPVA MNs (data not shown).

Data gathered during the characterization showed that the insulin did not cause any significant differences to the structural properties of the PVPVA needle layer. This established a physical baseline for the MNs before further investigating additional properties and understanding how adjustments to the backing layer formulation may adversely or positively affect the insertion profile.

### 3.4 Evaluation of Different Materials for the Backing Layer in MN Arrays

Figure 1 demonstrated that the choice of backing layer had a marked effect on the insertion of the MN array, however the material properties attributable to this have not been explored previously. Figure 3 (i) shows the toughness, hardness and flexibility of two of the backing layer formulations used throughout out this work, featuring Na-CMC and PVA as the main components.

**Figure 3:**
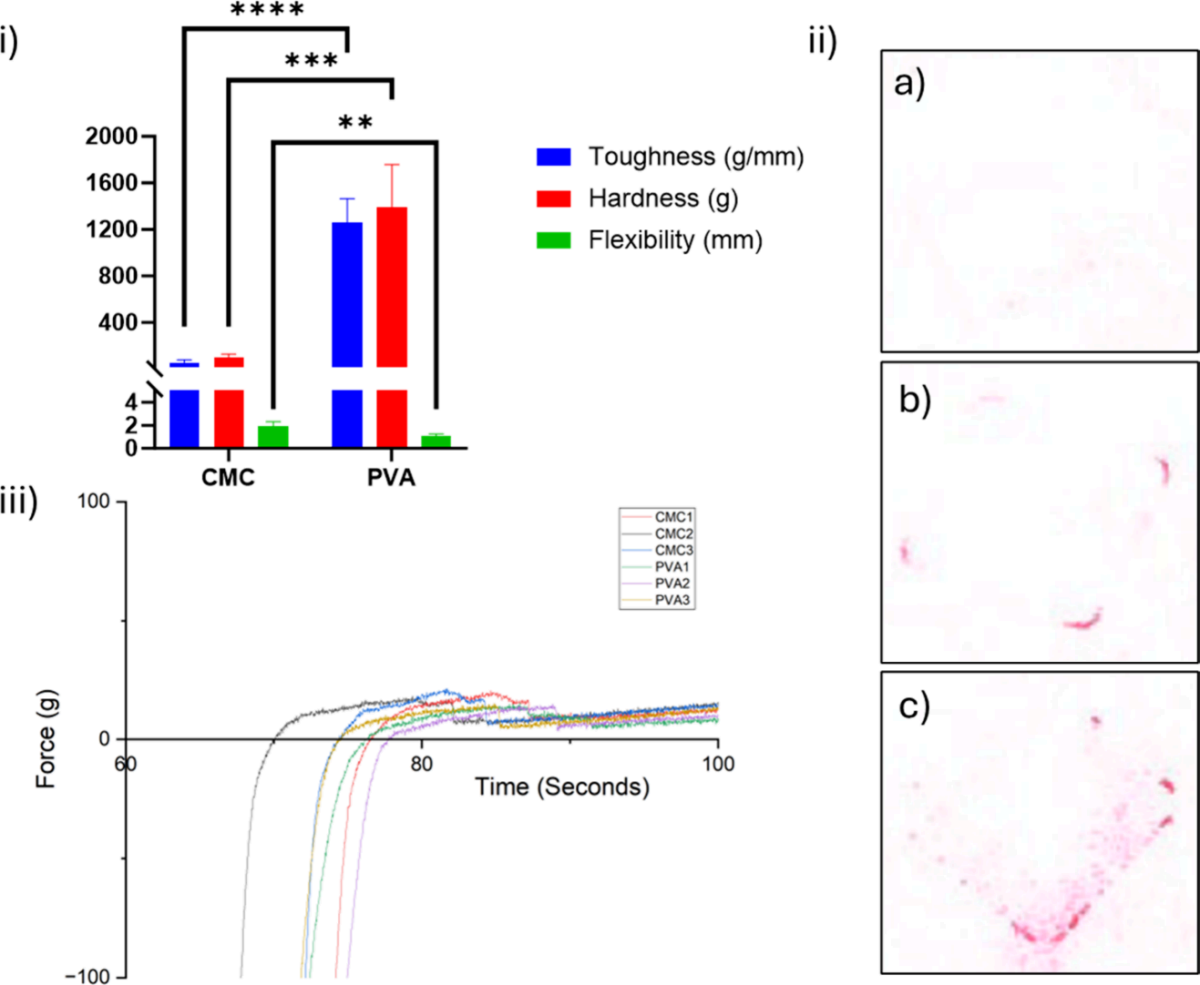
Graph (i) shows measurements of material properties for the Na-CMC and PVA formulations used for the MN array backing layers. Statistical significance was observed between the materials for all characteristics tested. *P* < 0.0001 (****), *P* < 0.001 (***) and *P* < 0.01 (**) were calculated for toughness, hardness and flexibility, respectively. Data expressed as *n* = 6, mean ± SD. Figure (ii) shows images of pressure-sensitive indicating film after MN arrays with backing layers (a) Na-CMC, (b) PVA, or (c) PS were applied to ex vivo porcine skin. Graph (iii) displays the force required to remove MN arrays with either a Na-CMC or PVA BL from porcine skin. Data expressed as *n* = 3.

The backing formulation containing predominantly Na-CMC had statistically significantly different measurements in toughness, hardness and flexibility compared to the PVA-based backing layer. It is likely that the flexibility of the Na-CMC was underreported as the sample failed to break under pressure, instead it became dislodged from the rig. Indeed, Na-CMC was selected for use as a backing layer material owing to its inherent flexibility, which may aid handleability and application.^26^

The backing layer made with 20% w/v PVA was less flexible than Na-CMC but both tougher and stronger. This suggests that the material the backing layer is made from may benefit from a high toughness and hardness, supporting the MNs across the whole surface area of the array to insert. Increased flexibility may have an opposite effect, reducing the overall insertion efficiency.

In an attempt to visualize the insertion of the MNs and how the material properties might affect this, pressure-sensitive indicating film (PSIF) was used. Figure 3 (ii) shows the imprint that MN arrays made on PSIF using PVPVA MNs with either a Na-CMC, PVA or PS BL. Despite the Texture Analyzer applying the same force, there are clear distinctions between the different materials. In each instance, the outline of the backing layer is the most definitive feature present. This was unexpected as the needles encounter the PSIF before the backing layer. Moreover, the imprint left by the PVA is much more distinct than Na-CMC, which could relate to the toughness and hardness of the material. Given the PSIF film responds to increased pressure to develop the imprint, this suggests that PVA exerted a larger force on the skin than Na-CMC, which is barely visible. This transfer of force may be an important factor for improving the insertion of the needles into skin. Interestingly, it may be that the flexibility of the Na-CMC layer plays a negative role in this, as the film is more likely to bend under pressure, rather than uniformly interface the skin. As per the section 2.2.1, glycerol is added to the Na-CMC backing layer to act as a plasticizer and aid removal from the PDMS mold. While improving the flexibility of the MN backing layer, and allowing the MN to withstand manipulation, this may have reduced the hardness of the formulation, thereby leading to the poor insertion observed here. Of the three MN arrays tested, PS showed the clearest outline however the material properties were not calculated owing to the previous failure in MN manufacture. Results from the PSIF may suggest that if the manufacturing method could be optimized, the insertion profile may be further improved with the use of a PS backing layer.

Later, an adhesion study was conducted to learn whether changing the backing layer material affects how successfully the MN array can adhere to skin once the MNs are inserted.

Figure 3 (iii) shows the force required for MN arrays Na-CMC and PVA to be removed from the skin after a predefined force is applied to the array for 1 min. The highest value for force was interpreted as the force necessary to successfully remove the MN from the skin, which was followed by a characteristic drop in force immediately thereafter. Upon first inspection the profiles of both MN arrays appear similar, however, the mean average forces are 0.20 and 0.15 N respectively, suggesting Na-CMC has a stronger attachment to the skin.

Frequently discussed by those within the field is the ability of MNs to remain inserted in the skin as a patient continues with their day-to-day life, particularly when the MN array is intended for prolonged release of a drug. Previous work by Muresan et al. showed that a Na-CMC backing layer adheres well to brain tissue, reducing the risk of dislodgement and helping to ensure complete drug delivery from the MNs.^27^ The data generated here may suggest that this is also the case with a Na-CMC backing layer despite the application being to skin.

Here, a difference in the force required for removal was observed after applying static force for 60 s. The force required for removal may increase if the application time is increased, due to the increased uptake of moisture from the skin surface, causing the backing layer to become more gel-like. In particular, this will likely be the case with Na-CMC given its polysaccharide structure and known bioadhesive properties. The application time will be dependent on the polymer dissolution speed and the corresponding drug release.

From a translational perspective, how to best protect the backing layer should be taken into consideration early on in the design of the product and packaging.^28^ Clearly, there is a need to ensure water content within the packaging is controlled to avoid the backing layer becoming unusable. Additionally, once the patient applies the array to their skin, how the MN array, including the backing layer, will be protected and not accidentally damaged must be carefully considered.

Having identified that the formulation of the backing layer had a significant effect on the insertion profile, understanding whether the backing layer influenced drug delivery required exploration. While the addition of insulin itself was not anticipated to have a negative impact on the MN insertion, the effect of varying the backing layer formulation on the therapeutic activity of insulin within the MN required exploration.

### 3.5 Insulin Localization and Stability within MN Arrays

As well as affecting the insertion profiles, it was hypothesized that the backing layer material may affect the localization of insulin within the MN array. Following on from earlier studies, investigation was required to understand whether the addition of an aqueous backing layer caused insulin to migrate away from the needle layer. To do this, insulin molecules were fluorescently tagged, allowing visualization.

Figure 4 shows the three MN array formulations loaded with FITC-insulin, as well as a blank MN (no insulin) (Images D), I) and N)) and the MNs with recombinant insulin loaded (Images E), J) and O)). Both the blank MN and untagged insulin loaded MNs show there is not any notable autofluorescence.

**Figure 4:**
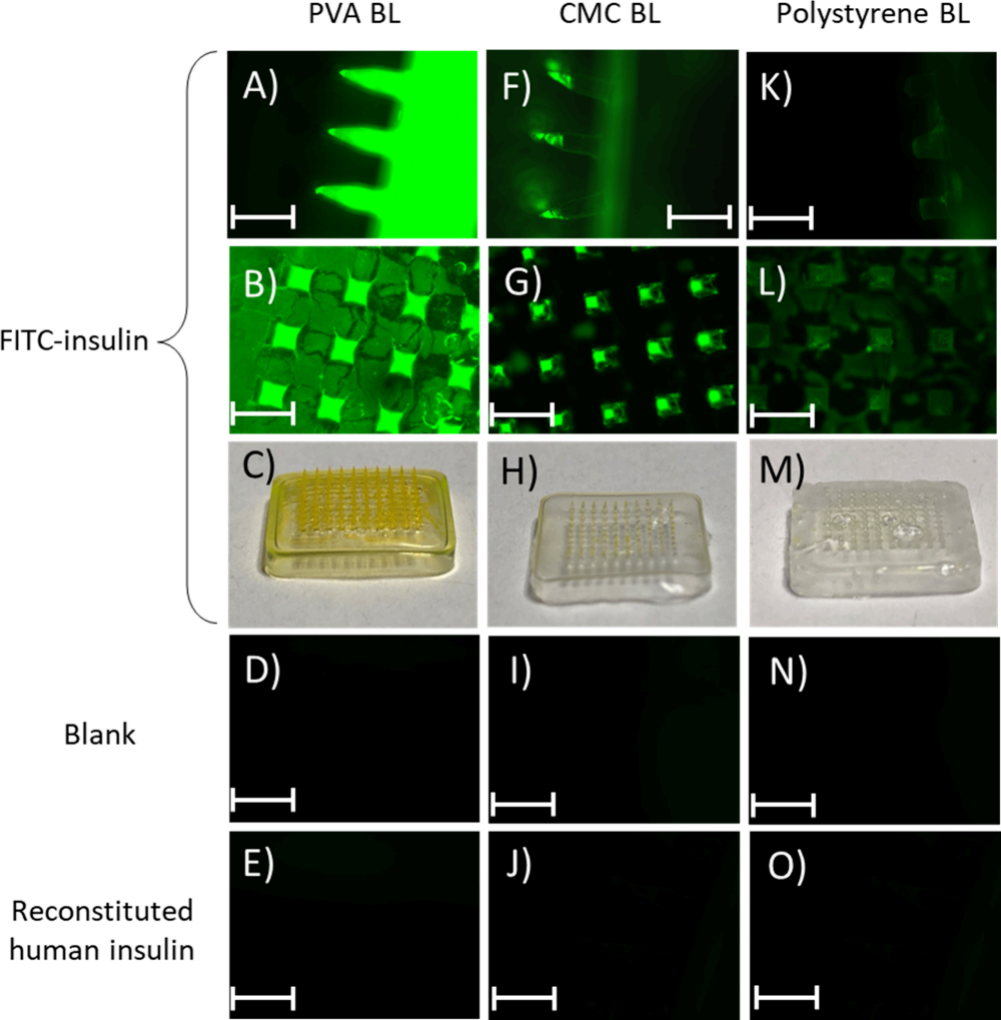
Images of three different MN formulations loaded with FITC-insulin. Images (A)–(C) show MNs with a PVA BL, images (F)–(H) show MNs with a Na-CMC BL, and images (K)–(M) show MNs with a PS BL. Images (D), (I), and (N) show blank MNs with the same composition material composition. Images (E), (J), and (O) show the comparable MNs loaded with human recombinant insulin. Gain was set to 0.002 on the fluorescent microscope. Scale bar is equivalent to 750 μm.

The images of MNs with a PVA BL show that FITC-insulin is present throughout the entirety of the MN, including the backing layer, despite only being loaded during the first step of manufacturing, when the needle layer is cast. Indeed, from C) alone it is clear to see this as both the needles and the backing layer have a yellow color associated with the presence of the FITC. This suggests that the FITC-insulin has migrated throughout the MN, potentially during the drying process. As mentioned previously, this will more than likely present issues with the delivery of insulin, including dosing accuracy.

In 2021, a simulation study was published by Feng et al. which modeled the diffusion of insulin in MNs made from PVA.^29^ The modeling demonstrated that PVA would interact with insulin through hydrogen bonding, although to a lesser extent that might be observed with other materials such as hyaluronic acid. Furthermore, PVA and insulin had a negative binding energy, suggesting repulsion between the two molecules. Given the homogeneity of insulin throughout the MN array, it does not seem the repulsive force of PVA has had a significant effect here.

PS was selected for its hydrophobic nature. Instead of using water as a diluent, 1,4-dioxane was used to dissolve the PS into solution. Given the absence of water in this MN array, it was hypothesized that the insulin may remain in the needle layer only. This concept was supported by work completed by Ziesmer et al., who compared the use of a water-soluble PVP backing layer to a poly­(methyl methacrylate) (PMMA) backing layer.^30^ The study demonstrated that when the PMMA backing layer was used sulforhodamine B dye, and subsequently the drug of interest, would not readily diffuse into the water insoluble backing layer. Despite this, although not as pronounced, the FITC-insulin appears to be uniformly dispersed throughout both the backing and needle layers of the array. However, it may be that the addition of the 1,4-dioxane unintentionally solubilized the insulin, facilitating the movement of insulin into the BL.

Quite oppositely, MN arrays featuring a Na-CMC BL demonstrated precise localization of the FITC-insulin at MN tips. Images F) and G) visualize these findings, showing that the FITC-insulin is present in each MN tip but not present in the backing layer. Image H) further shows the MN array is colorless except for the tips of the needles. The mechanism behind this is not fully understood; however, it could be attributed to insulin aggregating and settling at the bottom of the PDMS mold during centrifugation. Further investigation would be required prior to therapeutic use as for insulin to exert a physiological action it must bind to receptors in its monomeric form, rather than an aggregate.

The main advantage with ensuring insulin is only in the tip of the MN is that the full dose of insulin may be delivered even when the MNs do not fully insert or penetrate deep into the skin. As mentioned previously, when initial characterization was completed, MNs with a Na-CMC BL were only able to insert to 162.5 μm, notably less than the MN length, yet with the insulin so densely packed into the tip of the MN, this issue may be negated.

FT-IR was used to assess whether the FITC-insulin remained stable, essential for physiological activity. Figure SI3 shows the spectra of the different MN formulations loaded with insulin, compared to lyophilized FITC-insulin for reference. Immediately, amide I and II peaks can be identified in MNs with either a Na-CMC or PS BL, while it is less clear in the spectrum for the MNs with a PVA BL. Evidence of peak shifting is present in these spectra which could indicate changes to the insulin secondary structure.^31^

This work has demonstrated that choice of backing layer has a pronounced effect on the distribution of drug in the MN array. MN arrays with PVA or Na-CMC backing layers were progressed, first in an ex vivo insertion study and later a permeation study using ELISA for insulin detection.

### 3.6 Comparative *Ex Vivo* Drug Release and Permeation Study between MNs with Different Backing Layers

Figure 5 shows cross sections of skin which had a MN loaded with FITC-insulin inserted into it. MNs with the PVA BL appear to disrupt the surface of the skin, highlighted by the red circles showing regions where the stratum corneum is not continuous in Figure 5 B) and C). However, there is considerable fluorescence along the surface of the skin section. This is likely due to the presence of the FITC-insulin in the backing layer, which transferred to the skin surface when the needles were inserted.

**Figure 5:**
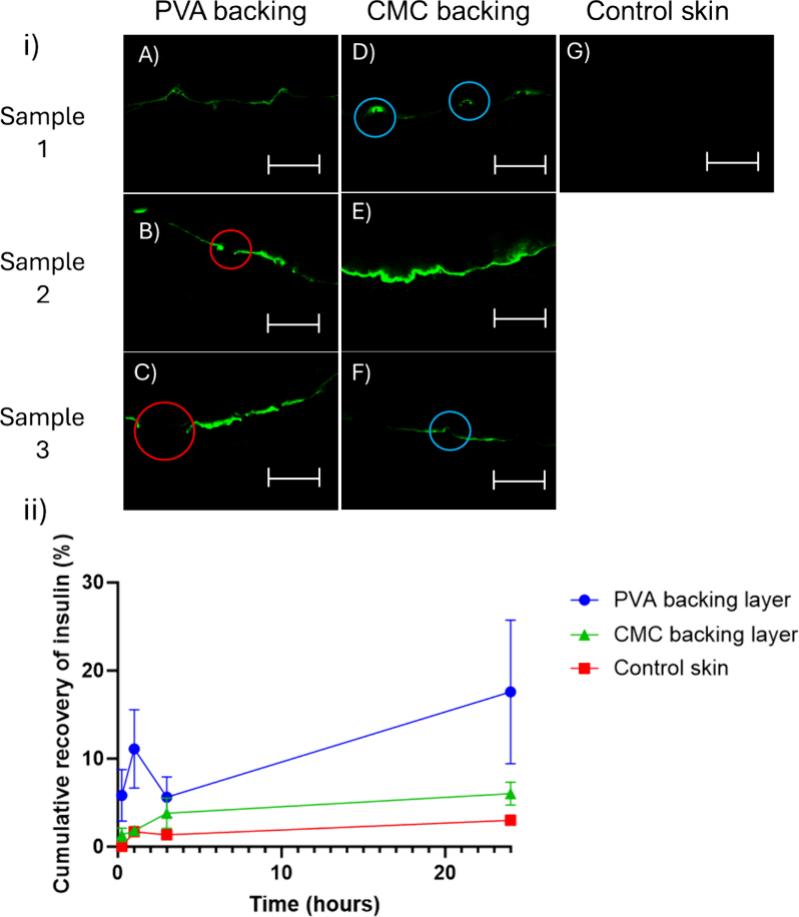
Image (i) shows ex vivo porcine skin sections under a fluorescent microscope after the manual insertion of MNs loaded with FITC-insulin. Images (A)–(C) are MNs with a PVA BL, (D)–(F) are MNs with a Na-CMC BL, and (G) is control skin with no MN insertion. The red circles highlight changes in the skin surface that are consistent with successful MN insertion, whereas blue circles show MN indentations. Skin sections were orientated so that the dermal layer of the skin aligns with the top pane of each image. Gain = 0.017, *n* = 3. Scale bar is equivalent to 750 μm. Graph (ii) shows the cumulative recovery (%) of insulin from receptor fluid in Franz diffusion cells at 15 min, 1 h, 3 h, and 24 h during a permeation study using MNs with either a Na-CMC or PVA BL 0.4% w/v insulin loaded MNs in ex vivo porcine skin. Data expressed as mean ± SEM, *n* = 6.

In comparison, images D) and F) show small regions along the skin surface that have a more intense fluorescence (shown in the blue circles). This corresponds to the FITC-insulin only being present in the tips of the MNs with a Na-CMC BL, which would be transferred to the skin upon insertion and dissolution. Where the fluorescence is not visible, in the deeper layers of skin, may suggest that the FITC-insulin has diffused as the MNs have dissolved. Alternatively, if the skin has not been successfully punctured, the FITC-insulin may have accumulated on the deflected skin surface.

An *ex vivo* permeation study was conducted using porcine skin as a human skin model in a Franz diffusion cell setup to quantify which MN formulation was superior in terms of insulin delivery.

Figure 5 (ii) gives the cumulative insulin recovery observed at four different time points over the course of 24 h. From the first time point taken after 15 min and throughout the remainder of the time course, MNs with a PVA BL have an increased insulin release compared to those with a Na-CMC BL. By 24 h, MNs with a PVA BL had released 17.6% of the loaded insulin, compared to 6.0% from those with a Na-CMC BL, almost three times less. Additionally, it may appear that the control samples have been contaminated with insulin, accounting for the raised values observed throughout the experiment, however, this is more likely due to nonspecific binding of proteins present in the *ex vivo* skin to the capture antibody used in the ELISA. This kind of interference is not uncommon when working with biological samples but may mean that the true values for insulin recovery from the MNs are lower than reported here.

Mentioned earlier was the possibility of insulin aggregation within the MNs with a Na-CMC BL. Although not confirmed, such aggregation could support the finding of reduced insulin recovery with this MN formulation.^32^ Aggregation would lead to reduced diffusion owing to an increase in molecular weight. Another explanation for the reduced recovery for both MN formulations could be adsorption of insulin to the experimental equipment.^33^ While adsorption cannot be discounted, where possible, MNs were prepared and tested using the same equipment and methodology, therefore standardizing the effect that adsorption might have. As such, it is anticipated that the difference between the recovery rates of insulin is due to the MN formulation.

When MNs with a PVA BL were tested a higher proportion of MNs insert into the skin to a deeper depth, as demonstrated in Section 3.1, releasing more insulin. However, it was hypothesized that having the insulin localized in the MN tips may improve the release profile of insulin however this is reliant on the MNs being able to puncture the skin, creating a channel for the insulin to diffuse through. As noted previously, when Na-CMC was tested, the MN arrays had a lower insertion efficiency, which may account for the low insulin recovery in the receptor fluid. It should be considered that some insulin will have remained in the skin, which was not quantified in this experiment.

Despite many researchers appearing to test their optimized MN formulations during *in vivo* studies, several published *ex vivo* studies have observed complete insulin release within a shorter time frame than was studied here. Work by Ling and Chen states that close to 100% of insulin was released from MNs manufactured from starch/gelatin within 4 min of insertion into porcine skin (insulin loading in the needle layer only).^34^ Despite the fast release observed in *ex vivo* studies, the corresponding *in vivo* studies failed to show peak plasma glucose levels until 2 h after insertion into the skin. This may be attributed to the methodology used to quantify the insulin, which quantified the amount of insulin on the surface of the skin before assuming the remainder was within the skin itself. Given this method, it would have been impossible to predict the time required for the insulin to pass through the skin, explaining when the subsequent drop in *in vivo* blood glucose levels happened so much later than originally anticipated. A further study by Yu et al. demonstrated >90% insulin release from modified alginate/hyaluronate MNs within 6 h using a similar method to that as Ling and Chen.^35^ Here, rat skin was used, which has considerably different physiological properties compared to human skin. Rat skin is known to be thinner than porcine and human skin, which could cause the insertion profile to appear vastly improved and give an artificially high insulin release.^36^ Lee et al. quantified the release of insulin from MNs made from gelatin with a 90 kDa Na-CMC backing layer using a Franz diffusion cell setup with *ex vivo* porcine skin, quantifying insulin in the receptor fluid with an ELISA.^37^ In this work a burst release was observed, whereby almost 50% of the loaded insulin was released in the first hour, increasing steadily to 83.4% at 5 h.

During early characterization studies, it was anticipated that the PVPVA MNs would take close to 24 h to fully dissolve when inserted into porcine skin (data not shown). The results presented here suggest that more time will be needed, indicating a slower rate of dissolution compared to other MNs tested in the literature. However, this may be an artifact of the incomplete insertion of the MNs, as the MNs require the interstitial fluid to dissolve, meaning they must have successfully punctured the skin first. Although unlikely to account for such dramatic variations, consideration should also be given to variation in porcine skin samples, including storage and handling between different research groups.

An explanation for a low insulin release in MN arrays with a PVA BL may come from the instability of insulin. Given ELISAs utilize antibodies for protein detection, the secondary structure of the protein being quantified must remain in its stable native state to allow for binding. The potential structural changes observed in MNs with a PVA BL may have had such a significant effect that the insulin no longer binds as successfully with the capture antibody, giving a lower absorbance readout at the end of the assay compared to the quantity of insulin that was present in the sample. More likely the case with PVA, insulin that is present in the backing layer is unlikely to have moved through the entirety of the MN array and enter the skin. Therefore, if a considerable amount of insulin is present in the backing layer this may account for the low overall recovery.

The data suggests that neither formulation gave complete insulin release from the MN array during the 24-h time frame measured, however a difference in insulin release was observed when different backing layer formulations were employed. Quantification of insulin remaining in the backing layer of the MN array and in the skin may further explain the low recovery.

## 4.0 Conclusions

Both Na-CMC and PVA are commonly used polymers in the MN field. Despite this, the data presented here demonstrated that using either Na-CMC or PVA in the BL of MN arrays can produce statistically significant differences in the insertion, and subsequent delivery, of insulin into skin.

This work attempted to explore and understand more about the material properties of both Na-CMC and PVA. While crude relationships may be observed between the materials tested in this study, further research is required to gain a fuller understanding. At this time, the authors are not aware of research that has specifically focused on relationships between flexibility, hardness and toughness, and how these may affect the insertion of MNs in human skin. Understanding and exploiting these relationships is an important avenue of research to aid the clinical translation and regulatory acceptance of MN arrays for medical use.

Building on this further, an optimized backing layer will likely need to be tailored for the proposed site of administration, meaning both the material and the associated properties will require rational selection and justification. Throughout this work, the focus remained on insertion of MNs into skin. Previous work has investigated the feasibility of using MNs in the eyes or brain, tissues which both have vastly different properties compared to skin. As such, it is likely that different degrees of flexibility, toughness and hardness will be required for the successful insertion of MNs in different target tissues. Future work should also build on the findings described in Section 3.5, which highlighted the possibility of tip-loading insulin with a Na-CMC BL. It would be of value to explore these findings with several different drug molecules.

Overall, differences observed between the insertion of MNs and associated drug delivery when a range of backing layer formulations were employed, suggests that the backing layer plays a critical role. These findings play a key part in ensuring that MNs can be reproducibly and consistently inserted, advancing toward the successful clinical translation of MNs.

## Supplementary Material



## Notes

Two-step casting method, compression force-displacement graphs, and FT-IR spectra of lyophilized FITC insulin and FITC insulin-loaded MNs with differing backing layer formulations (PDF)
