# Supplementary material for: Optimisation of Backing Layer Formulations via Rational Polymer Selection to Improve the Insertion of Dissolving Microneedles Into Skin
Source: Mol Pharm. 2026 Jan 9;23(2):757–67. doi: 10.1021/acs.molpharmaceut.5c01024 (PMC12869474; doi:10.1021/acs.molpharmaceut.5c01024)
Supplement: Supplementary file 1 [file mp5c01024_si_001.pdf]

Supporting information: Optimisation of backing layer formulations via rational polymer selection to improve the insertion of dissolving microneedles into skin

Authors: Fiona Smith[a†], Benjamin Fiedler [a††], Khaled Elkassas [b], Ruslan Mohamed[a], Karmen Cheung[c], Mischa Zelzer [a], Abina Crean [b], Faz Chowdhury[c], Joel Segal[d], Frankie Rawson[a] & Maria Marlow[a]*

[a] School of Pharmacy, University of Nottingham, Nottingham NG7 2RD, United Kingdom

[b] School of Pharmacy, University College Cork, Cork, T12 YT20, Republic of Ireland

[c] Nemaura Pharma Limited, Advanced Technology Centre, Oakwood Drive, Loughborough, Leicestershire LE11 3QF, United Kingdom

[d] Department of Mechanical, Materials and Manufacturing Engineering, Faculty of Engineering, University of Nottingham, Nottingham NG8 1BB, United Kingdom

[†] School of Health and Life Sciences, Teesside University, Middlesbrough TS1 3BX, United Kingdom

[††] School of Pharmacy, University College London, London, WC1N 1AX, United Kingdom

*Corresponding author: Maria.Marlow@nottingham.ac.uk, Tel.: 0115 8467045

Two-step casting method

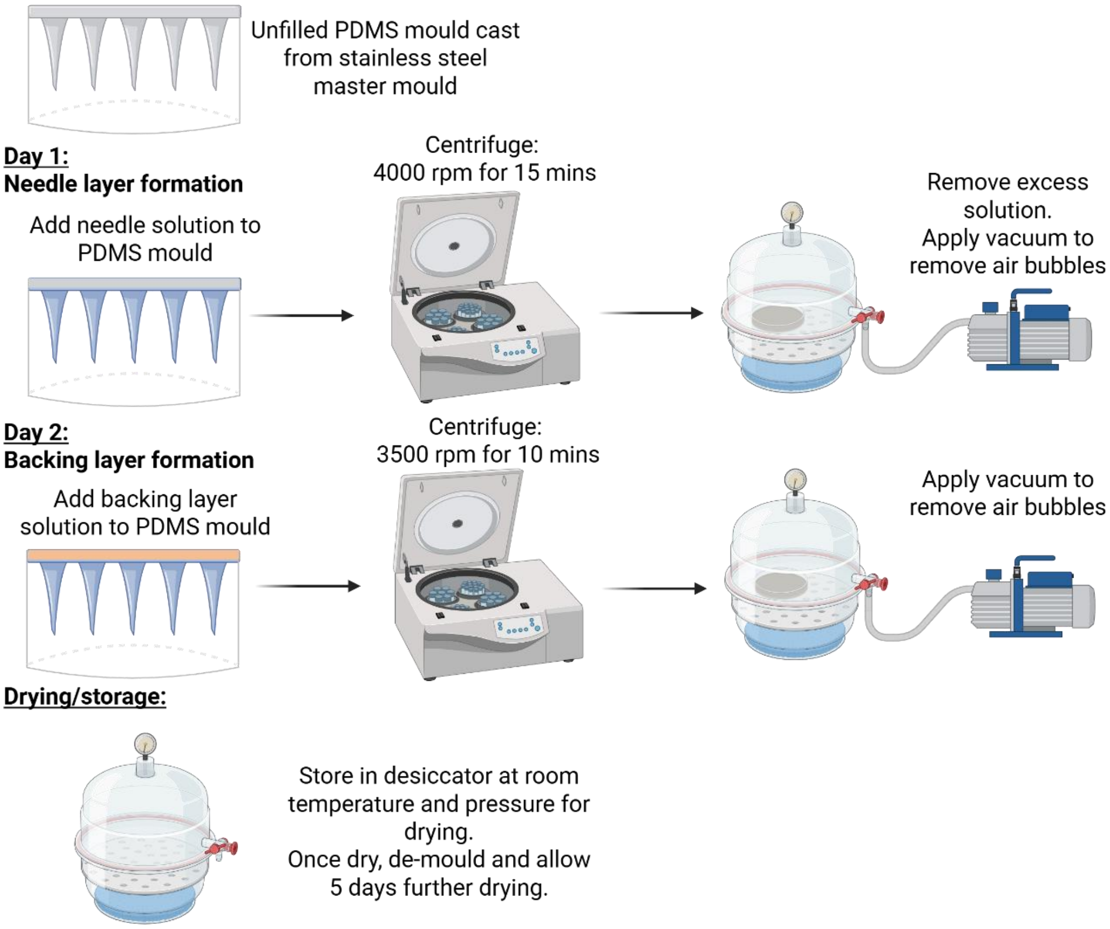

Figure SI1: *Schematic of the process to create microneedle arrays, in which the needle layer formulation is added on Day 1, centrifuged, and held under a brief vacuum. Then on Day 2 a backing layer, made from an alternative formulation, is added, centrifuged, and again exposed to a short vacuum, before being left for further drying at room temperature and pressure. This process is referred to as a two-step casting method.*

Compression force-displacement graphs

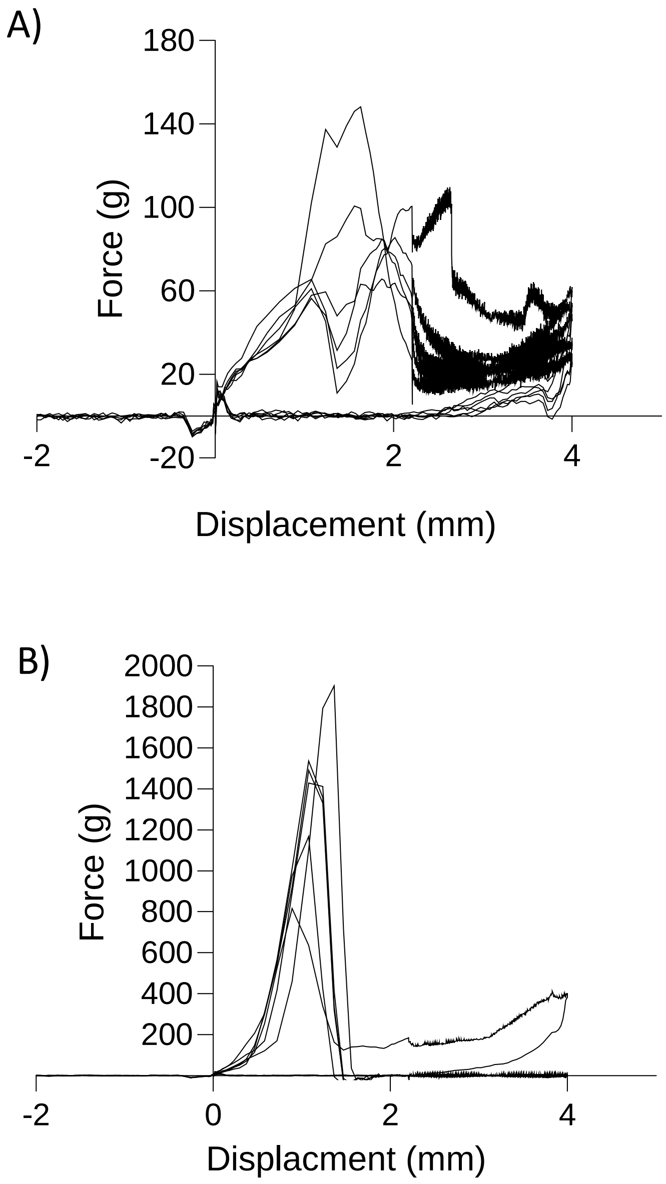

Figure SI2: *Compression force displacement graphs for A) Na-CMC and B) PVA backing layer formulations. Solutions were cast into PDMS moulds of the same geometry and volume as the MN array backing layer. Samples were tested using a three-point bend rig fixed to a texture analyser. Raw data was analysed to give hardness, flexibility and toughness measurements, n=6.*

FT-IR spectra of lyophilised insulin and insulin-loaded MNs with differing backing layer formulations

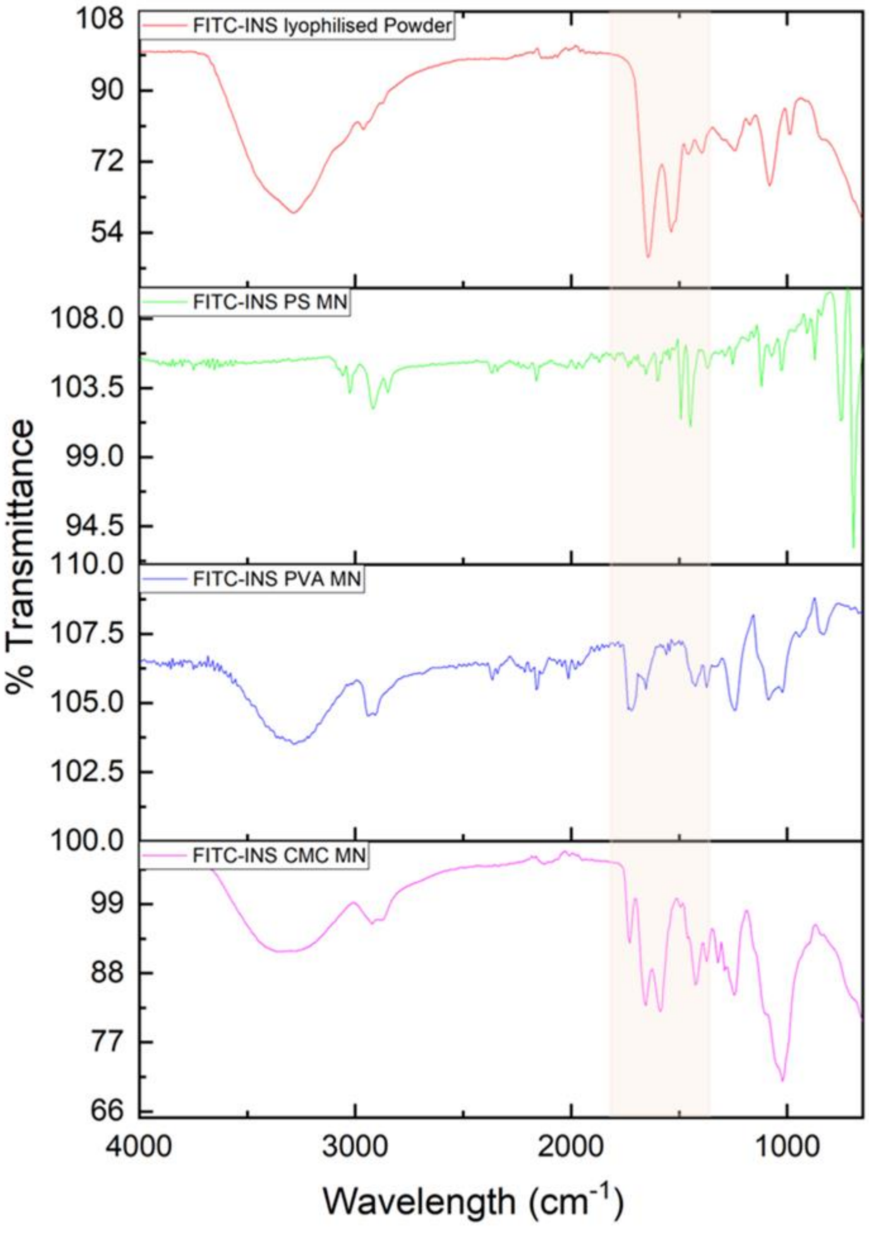

Figure SI3: *FT-IR spectra of lyophilised FITC-insulin compared to FITC-insulin loaded MNs with either a Na-CMC, PVA or PS BL. The amide I and II peaks, characteristically identifiable within the highlighted area (1400 – 1800 cm*$^{-1}$*), are well-defined when Na-CMC or PS is used in the backing layer, however there are deviations when PVA is used.*
